# Emerging role of G9a in cancer stemness and promises as a therapeutic target

**DOI:** 10.1038/s41389-021-00370-7

**Published:** 2021-11-13

**Authors:** Joshua R. Haebe, Christopher J. Bergin, Tamara Sandouka, Yannick D. Benoit

**Affiliations:** Department of Cellular and Molecular Medicine, University of Ottawa, Ottawa, ON K1H 8M5 Canada

**Keywords:** Cancer stem cells, Epigenetics, Phenotypic screening

## Abstract

The histone methyltransferase G9a is well-documented for its implication in neoplastic growth. However, recent investigations have demonstrated a key involvement of this chromatin writer in maintaining the self-renewal and tumor-initiating capacities of cancer stem cells (CSCs). Direct inhibition of G9a’s catalytic activity was reported as a promising therapeutic target in multiple preclinical studies. Yet, none of the available pharmacological inhibitors of G9a activity have shown success at the early stages of clinical testing. Here, we discuss central findings of oncogenic expression and activation of G9a in CSCs from different origins, as well as the impact of the suppression of G9a histone methyltransferase activity in such contexts. We will explore the challenges posed by direct and systemic inhibition of G9a activity in the perspective of clinical translation of documented small molecules. Finally, we will discuss recent advances in drug discovery as viable strategies to develop context-specific drugs, selectively targeting G9a in CSC populations.

## Introduction

**Fig. 1:**
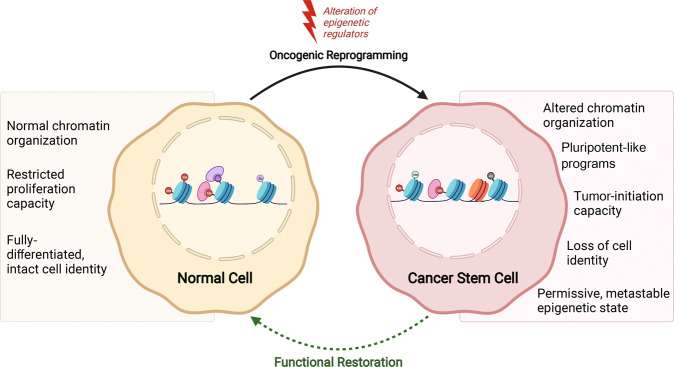
Transformation-acquired epigenetic signature mediating the cancer stem cell phenotype. Misregulation of epigenetic modifiers is commonly observed in cancer and drives oncogenic reprogramming of healthy tissues cells (left) into CSCs (right). Therapeutic strategies targeting the CSC epigenome, via inhibition of key epigenetic regulators aim to block the biogenesis of CSC via cellular plasticity, to restore normal-like functions such as differentiation and apoptosis sensitivity.

Experimental evidence in both leukemic and solid malignancies supports a hierarchical organization of tumor cell heterogeneity, in which cancer initiation and dissemination capacities are restricted to rare subpopulations of cancer stem cells (CSCs) [1–4] (Fig. 1). Key defining characteristics of CSCs, such as self-renewal functions and a tumor-initiating capacity have been extensively documented through in vivo serial transplantation assays, providing robust measures of both properties in limited fractions of bulk tumor mass [2, 5, 6]. More recently, lineage-tracing experiments and barcode sequencing have further increased our knowledge of CSC plasticity and clonal diversity [7–9]. It is now becoming clearer that the CSC phenotype holds a metastable state determined by non-mutational chromatin rearrangements and microenvironmental cues [10, 11] (Fig. 1). With the ability to maintain an equilibrium with early tumor progenitor cells via cellular plasticity, CSC populations can be restored following therapy to reinstate tumor growth and disseminate at distant organ sites [9, 10]. This phenomenon is accompanied by the acquisition of resistance mechanisms that restrict subsequent therapeutic options in the clinic [10, 12, 13]. Therefore, CSC populations represent the major clinical obstacle that remains unaddressed by conventional therapeutic measures [12, 13].

Molecular parallels between pluripotency and cancer stemness have been established by multi-omic investigations, revealing a shared molecular network between human pluripotent stem cells and CSCs [14–16]. As described for pluripotent reprogramming [17–19], advances in cancer epigenetics show that chromatin rewiring is essential to the emergence of CSCs by promoting self-renewal capacities [11, 20–23] (Fig. 1). This supports earlier observations of a distinct epigenetic signature, linking oncogenic DNA methylation to embryonic stem (ES) cell-like histone methylation patterns in human tumors [24]. Such a signature differing from healthy stem/progenitor and bulk tumor cells is sought to promote self-renewal and tumorigenic properties [24–26]. Notably, an aberrant function of the DNA methyltransferase DNMT3A, caused by a recurrent, somatic R882H mutation in hematopoietic stem cells was identified as a key event in the emergence of pre-leukemic hematopoietic stem cells, and the subsequent onset of acute myeloid leukemia (AML) [27]. Thus, profound changes in DNA methylation are characteristic of both early oncogenic transformation [28], and induced pluripotent reprogramming through forced expression of OCT4 and SOX2 [29, 30]. Consistently, transient expression of pluripotent reprogramming factors, resulting in premature/altered iPSCs has been linked to cancer development in vivo [31]. Tumors arising from incomplete pluripotent reprogramming display important epigenetic alterations distinguishing them from normal tissue, and mainly including loci-specific DNA hypermethylation, global hypomethylation, as well as gene-specific dysregulation of histone H3 lysine27 trimethyl (H3K27me3) deposition [31] (Fig. 1). Several studies demonstrated that increased expression or enhanced activity of development-associated chromatin regulators, such as the Polycomb group (PcG) proteins, is tightly linked to CSC development and maintenance [32–36]. Considering parallels between CSCs and pluripotency, the concept of oncogenic reprogramming emerged in the literature as an interplay between transcription factors and chromatin regulators which is essential to sustain the CSC phenotype through dynamic cellular plasticity [11, 20, 37, 38] (Fig. 1).

## Histone modifications and oncogenic reprogramming

Covalent modifications of core histone tails are fundamentally connected to transcriptional regulation, by influencing the dynamic patterning of euchromatin (active) and heterochromatin (repressed) within the nucleus of eukaryotic cells [39]. While histone lysine acetylation generally facilitates transcriptional activity, the methylation of specific lysines and arginines can be associated with either repressed or active transcriptional states [39–41]. The impact of mono, di, and trimethylation of lysines, as well as mono, and symmetrical or asymmetrical methylation of arginines on transcriptional activity is influenced by the position of the target residue(s) and the presence of other cooperating epigenetic marks [39, 41]. The mutation or deregulation of enzymes catalyzing the deposition (writers) or removal (erasers) of histone marks is crucial to either promote or block the pluripotency state and can represent driver events of CSC biogenesis. One of the most documented examples applicable to both concepts is the histone methyltransferase (HMTase) and Polycomb repressive complex-2 (PRC2) member EZH2 [32, 34, 36, 42].

### The HMTase G9a as a major epigenetic regulator in embryogenesis and cancer

In addition to PcGs, several regulators of histone H3 lysine-9 (H3K9) methylation were recently suggested as key actors in oncogenic reprogramming, based on their role in pluripotency and specific malignancies [20, 43–48]. Among these, the SET domain-containing histone methyltransferases SUV39H1, SETDB1, and G9a (EHMT2), which play distinct roles in the maintenance of H3K9 methylation states and the organization of heterochromatin [49], are gaining much attention in recent cancer literature, as reviewed by Saha & Muntean [50]. The case of G9a is particularly interesting, given its emerging role in the context of neoplastic stemness [21, 51–54].

**Fig. 2:**
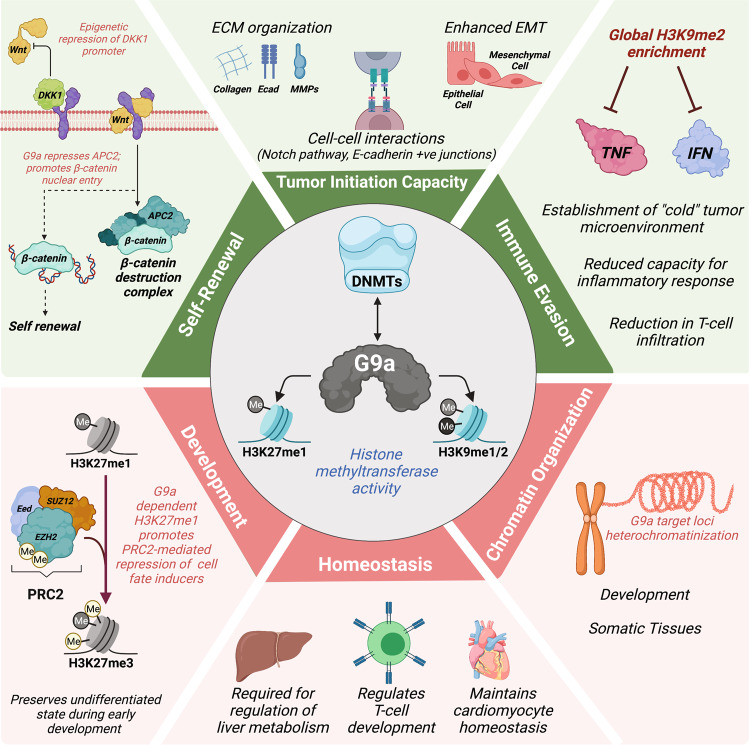
Context-specific roles of G9a in neoplastic and healthy tissues. In cancer stem cell populations (green), G9a functions at multiple levels to drive cancer progression through molecular networks maintaining self-renewal and tumorigenicity. By promoting the CSC phenotype, G9a contributes to tumor immune evasion. On the other hand, in healthy tissues (red), G9a is an essential regulator of cell fate and differentiation genes, homeostasis, and maintenance of heterochromatin. Although G9a was extensively linked to cancer progression, the untargeted inhibition of its HMTase activity may have deleterious effects on normal cell functions and compromise future translational applications.

Functionally, G9a, along with its partner GLP (G9a-like protein), selectively mono- and di-methylate H3K9 (H3K9me1/2) and has been extensively linked to the epigenetic regulation of pluripotency during early embryogenesis (Fig. 2) [55]. G9a was also suggested to mono-methylate H3K27 (H3K27me1), serving as a template for subsequent PRC2-mediated gene repression upon H3K27 di and trimethylation by EZH2 (Fig. 2) [56, 57]. While H3K9me2 is typically associated with transcriptional repression via passively blocking the deposition of activating acetylation marks on H3K9 [55, 58, 59] at euchromatic loci [55], mono-methylation of H3K9 is enriched at the promoters of transcriptionally-active genes [58]. In murine pluripotent cells, H3K9me2 was found to be significantly enriched at facultative heterochromatin domains, marking specific loci that can adopt either a further compacted conformation (constitutive heterochromatin) or revert back into transcriptionally-active euchromatinic regions [60]. Constitutive silencing of H3K9me2-marked regions can be achieved by the action of chromatin writers such as SUV39H1 and SETDB1 trimethylating H3K9 (H3K9me3), and/or via the recruitment of additional chromatin-associated repressors, such as HP-1 and MPP8, linking the H3K9 methylation state to DNA methylation machinery [61–65]. However, a complete loss of H3K9 methylation does not immediately lead to global DNA demethylation [49].

At the early onset of pluripotent cell priming, G9a was shown to catalyze the deposition of H3K9me2 at the promoters of pluripotency genes such as OCT4, ultimately promoting heterochromatinization via the recruitment of HP-1 and DNMT3A/B [43]. Similar studies have further solidified the role of G9a in regulating pluripotency, identifying it as a barrier to reprogramming [66, 67]. In contrast, G9a-mediated accumulation of H3K9me2 was shown to be crucial for driving the early postimplantation phase of embryo development, where it represses key developmental regulators independently of gene silencing exerted by H3K27me3 deposition (Fig. 2) [68]. Still, the catalysis of H3K27me1 by G9a, leading to PRC2 recruitment, supports additional roles for this SET-domain HMTase in preserving undifferentiated cell states in early development (Fig. 2) [57]. Beyond G9a HMTase function, chromatin-associated factors such as MPP8 were shown to participate in defining the G9a-dependent pluripotent transcriptional network. MPP8 can form a complex with G9a/GLP heterodimers and DNMT3A, contributing to the heterochromatinization of G9a target loci [65]. Recent work by Muller et al. described the methylation-independent role of MPP8, in conjunction with G9a, in the repression of pro-oncogenic LINE1 retrotransposon sequences in healthy pluripotent stem cells. While SUV39H1/H2 were previously thought to be implicated in LINE silencing, it is in fact G9a that is required to facilitate the recruitment of MPP8 to these elements [69]. In the context of oncogenic reprogramming, the action of G9a may contribute to rewire the epigenome of a neoplastic cell toward an aberrant trajectory, promoting self-renewal and blocking functional differentiation without achieving bona fide pluripotency. Furthermore, recent data characterizing its role as a molecular scaffold supports that G9a HMTase activity represents only a single facet of its regulatory repertoire in early development and cancer. Therapeutic strategies exclusively focussing on blocking H3K9me1/2 deposition may leave G9a able to interact with and recruit other important pro-oncogenic regulators.

Numerous studies reported overexpression or enhanced activation of G9a in various types of malignancies, with higher G9a abundance and enriched H3K9me2 deposition often correlated with poor clinical outcomes [21, 70–73]. Early investigation of G9a in the context of neoplasia revealed that knocking down G9a or inhibiting the deposition of H3K9me2 in human breast cancer cell lines causes the de-repression of specific tumor suppressor genes [74]. Then, oncogenic activation or overexpression of G9a was linked to the upregulation of the canonical Wnt pathway, which plays a central role in self-renewal and tumorigenesis (Fig. 2) [75]. Specifically, G9a was shown to epigenetically repress the promoter of Dickkopf (DKK) genes in renal and pancreatic tumor cells, consequently blocking the inhibitory effect of DKK proteins on the Wnt co-receptors LRP5/6 (reviewed by Benoit et al. [76]). This impact of G9a on the canonical Wnt pathway was further validated in melanoma, where the genetic or pharmacological suppression of G9a functions also downregulated canonical Wnt activity through DKK1 [71]. Such observations, together with findings supporting the role of G9a promoting proliferation, migration, and survival in different types of neoplasms [77–79] underscores its potential as an anticancer therapeutic target. This has nurtured a growing number of recent studies, particularly in the context of tumor heterogeneity and neoplastic stemness.

## G9a is a key regulator of neoplastic stemness

Beyond previous indications of G9a driving the bulk development of several neoplasms, its role as a regulator of key CSC functions, such as self-renewal and tumorigenicity, has been recently characterized. The foundation of this concept comes from a study by Lehnertz et al. depicting the essential regulatory role of G9a in maintaining CSC populations in hematological malignancies [52]. G9a expression was found to be increased in mouse hematopoietic progenitors, at similar levels to mouse ES cells, while low expression was observed in mature myeloid and lymphoid cells [52]. Conditional deletion of G9a in the mouse hematopoietic system did not yield significant changes in progenitor frequency and lineage commitment [52]. However, AML progression and the self-renewal activity of leukemic stem cells were greatly impaired in G9a deficient animals [52]. Reintroduction of an HMTase-dead mutant of G9a in knockout mouse AML cells demonstrated that the impact of G9a on self-renewal and differentiation blockade in leukemic stem cells is relying on H3K9me2 deposition [52]. Moreover, the inhibition of G9a HMTase activity using UNC0638 restored myeloid differentiation in murine AML cells [52]. Ultimately, this study concluded that G9a exerts its effect on AML stemness by promoting HoxA9-dependent transcription. Such a transcriptional network was extensively related to the maintenance of self-renewal and undifferentiated states in hematopoietic stem cells [52]. These findings were corroborated in solid tumors where G9a activity was shown to promote key functional features of CSCs, such as tumor-initiating capacity and epithelium-to-mesenchyme transition (EMT) in human colorectal cancer, non-small cell lung cancer (NSCLC), and head and neck tumor tissues (Fig. 2) [21, 53, 80].

### G9a regulates pluripotent transcriptional networks in cancer

Multi-omics and in silico studies highlight transcriptional parallels existing between pluripotent stem cells and CSCs. Analyzing the enrichment of gene sets associated with ES cells in different types of human cancers revealed that tumors presenting poor differentiation characteristics show enhanced expression of OCT4, SOX2, and c-Myc activated target genes [14]. Preferential repression of PcG target genes was also observed in poorly differentiated tumors. This pluripotent-like signature also correlated with poor clinical outcomes in human breast cancer [14]. Next, a study by Kim et al. established a multimodule c-Myc-dependent transcriptional network in ES cells as a tool to assess self-renewal and other CSC-associated functions in human neoplasms [15]. Recently, the concept of enriched pluripotent-like gene expression in CSCs was used to develop a machine-learning algorithm using the whole transcriptome of tumor samples to determine their individual degree of cancer stemness, or “stem cell index” [16]. Such an approach enables the assignment of a stem cell index to a myriad of individual tumor samples, from multiple origins, and which correlates with key aspects of CSCs such as oncogenic de-differentiation, metastatic progression, and tumor-infiltrating immune cells. An important link between the persistence of a pluripotent-like transcriptional signature and G9a activity was recently established in colorectal CSCs using transcriptional stem cell index attribution (Fig. 2) [21]. Using transcriptomic data from the TCGA human colorectal adenocarcinoma cohort (COAD), Bergin et al. established that tumors displaying an elevated stem cell index were also expressing high levels of G9a [21]. Pharmacological inhibition of G9a in the highly tumorigenic colorectal cell line HCT116 significantly decreased the expression of genes associated with pluripotency and restored markers of intestinal differentiation [21]. Interestingly, G9a inhibition in colorectal CSCs enriched from primary tumor samples showed a reduced tumor-initiating capacity in a serial organoid plating assay [21]. Similarly, G9a was found to promote in vivo tumorigenicity in NSCLC, and downregulation of H3K9me2 in vitro decreased the expression of CSC markers such as CD133 and CD44 [53]. In NSCLC, G9a maintains active Wnt signaling via the epigenetic repression of the gene APC2 [81], which represents another layer of H3K9me2-dependent regulation for this pathway, in addition to the control of DKK proteins (Fig. 2) [71, 76]. Considering the role of the Wnt pathway in maintaining pluripotency (reviewed by Sokol, [82]), these observations subscribe to the concept that G9a is intrinsically linked to ES-like transcriptional signatures in CSCs. However, one exception to the stem-promoting role of G9a was reported in lung adenocarcinoma, where its deletion or the inhibition of H3K9me2 deposition drove murine and human tumors toward a CSC-like tumor-propagating phenotype in vivo and in vitro [83]. The authors of this study claim that inhibiting lysine demethylases (KDMs) responsible for removing H3K9me1/2 would represent an approach to target CSC-like populations in advanced lung adenocarcinoma [83]. It is not clear, however, whether these are generalizable findings or only applicable to a distinct clinical subset(s) of lung adenocarcinoma. Still, it suggests that the benefits from G9a inhibition in CSCs may be patient and/or context-specific, which is an important aspect to consider in personalized medicine.

### G9a, CSCs, and the tumor microenvironment

An important aspect of CSC biogenesis resides in the complexity of the tumor microenvironment (TME), which is involved in dynamic crosstalk events with tumor cells to stimulate stem-like molecular programs and adaptive therapeutic resistance [10, 84]. This includes the modulation of key cell–cell interactions (e.g., Notch receptors) and extracellular matrix (ECM) elements, shaping the niche of CSCs and promoting the maintenance of self-renewal [85]. An integrative analysis combining ChIP-sequencing of G9a/H3K9me2 co-occupied genomic elements in patient-derived colorectal CSCs and transcriptome profiling upon G9a inhibition (UNC0642) revealed that G9a has an important role in regulating the expression of ECM elements, such as collagens and matrix-metalloproteinases (MMPs) (Fig. 2) [21]. The exact role of each identified ECM element impacted by G9a/H3K9me2 in CSCs has not yet been validated, but it is well-documented that cell–substratum interactions are critical to modulate pro-oncogenic functions such as differentiation, migration/invasion, and survival [86].

In addition to ECM elements, the TME encompasses the cellular composition of a tumor, aside from the neoplastic cells per se [85]. This includes cancer-associated fibroblasts, endothelial cells, and immune cells. Building upon previous reports linking Wnt signaling in tumor cells to immune evasion [87], Kato et al. established that oncogenic activation of G9a in human melanoma fosters the establishment of an immunologically “cold” TME, demonstrating significant reductions in T-cell signatures [71]. Moreover, they confirm that G9a inhibition using the small molecule UNC0638 was able to restore an immune-sensitive TME in vivo, and enhanced melanoma’s response to immune checkpoint inhibitors (CTLA-4 and PDL-1) in combinatorial treatments [71]. An independent study by Kelly et al. also confirmed that G9a suppression was effective at broadening the proportion of melanoma patient samples responding to immune checkpoint inhibitors (Fig. 2) [88]. It is now becoming clear that immune cell exclusion in solid tumors is a characteristic of the CSC-like phenotype [89]. Indeed, CSCs tend to exhibit epigenetic repression of endogenous retroviral elements, TNF and type-I interferon signaling pathways, as well as promoting immunosuppressive cascades, which all contribute to immune evasion [89]. In addition to melanoma, chromatin silencing exerted by G9a was also related to similar mechanisms in colorectal and breast CSCs (Fig. 2) [21, 90]. While G9a activity is required to maintain DNA methylation-based silencing of LINE1 retrotransposons in highly tumorigenic colorectal cancer cells [21], the reactivation of such genomic elements was shown to promote viral mimicry, interferon responses, and immunogenic cell death in CSC populations [91]. In addition, recurrent breast tumors, heavily relying on CSC activity, display a rewiring of the histone methylome through enrichment of H3K9me2 and acquire a dependence on G9a compared to primary tumors [90]. This was associated with the repression of the TNF signaling pathway, effectively impairing the inflammatory ability of the tumor and facilitating evasion of circulating immune cells [90]. Considering the important relationship existing between G9a in CSCs and the TME, including the modulation of immune sensitivity, it is conceivable that G9a expression and H3K9me2 levels in tumors could eventually become a new biomarker to predict patient responses to immunotherapy in the clinic.

## Pharmacological targeting of G9a as a therapeutic strategy to eliminate CSCs

The obvious contribution of epigenetics in cancer initiation and progression attracted much attention to the “druggable” aspect of several chromatin editing enzymes, including G9a [50]. Consequently, several small molecule inhibitors of G9a HMTase activity were developed, such as the quinazoline core-based BIX-01294 [92], UNC0638 [93], and UNC0642 [94], which block the H3 substrate binding site of G9a [95] (Table 1). While BIX-01294 and its closest analogs showed high toxicity in cell assays, UNC0638 presented poor pharmacokinetics in vivo [95]. The optimal balance between functional potency (H3K9me2 inhibition) and pharmacokinetic profile for a quinazoline core-based inhibitor was obtained with UNC0642, a closely related analog of UNC0638 presenting improved half-life, intrinsic clearance, and maximum serum concentration in vivo [94]. It is noteworthy that H3 competitive inhibitors such as BIX-01294, UNC0638, and UNC0642 display similar potency for G9a and its closely related partner GLP [95]. The indole core-based molecule A-366 was also identified as another type of H3 peptide competitive inhibitor of G9a, with a higher selectivity for G9a over GLP (~10-fold) [95] (Table 1). A-366 has shown pro-differentiation effects on leukemia cell lines, sensitization to DNA double-strand break inducers in osteosarcoma, and reductions in tumor burden in vivo using xenograft models of leukemia [96, 97].

**Table 1:** Main direct inhibitors of G9a HMTase activity reported in the literature.

Compound name	Structure	Class of inhibitor	Selectivity, [Ref.]	Notable anticancer effects, [Ref.]	In vivo tested, [Ref.]	Clinical development status	Reported limitations to clinical development, [Ref.]
BIX-01294	Quinazoline core	H3 peptide competitive	G9a (2.7 uM) > GLP (38 uM), [92]	Colon [21], Breast [108]	Yes, [108]	Experimental	High cellular toxicity [92, 93]
UNC0638	Quinazoline core	H3 peptide competitive	G9a (<15 nM) > GLP (19 nM), [93]	Leukemia, [70], Prostate [93], Lung [99]	Yes, [99]	Experimental	Poor in vivo pharmacokinetic (PK) properties, [95]
UNC0642	Quinazoline core	H3 peptide competitive	G9a = GLP (<2.5 nM), [94]	Colon [21], Melanoma [89]	Yes, [89]	Experimental	Toxicity in normal plasma cells, [103]
A-366	Indole core	Non-SAM competitive	G9a (3.3 nM) >GLP (38 nM), [95]	Leukemia [96], Prostate [96]	Yes, [96]	Preclinical (Leukemia)	Unknown
CM-272	Quinoline core/Dual	H3 + DNA substrate competitive	G9a (8 nM) >DNMT1 (382 nM), [95]	Bladder [73], Leukemia [98], Lymphoma [98]	Yes, [73, 98]	Preclinical (Hematopoietic malignancies, bladder)	Unknown
DS79932728	Aminoindole core	Non-SAM competitive	G9a (12.6 nM) >GLP (75.7 nM), [101]	No	Yes, [101]	Preclinical (β-thalassemia and sickle cell disease)	Unknown
EZM-8266	Unknown	Unknown	Unknown	No	Yes, [Unpublished, Epizyme, Inc]	Preclinical (Sickle cell disease)	Discontinued due to preclinical toxicology concerns [101]

Considering the cooperation of G9a and DNMTs in oncogenically relevant gene silencing, efforts were also deployed to develop small molecules simultaneously blocking both chromatin writers’ function with the hope to get stronger anticancer effects (Dual inhibitors) [95]. The first reversible G9a/DNMT1 dual inhibitor that was reported in the literature is CM-272, a quinoline core-based molecule with high selectivity and in vivo bioavailability [98] (Table 1). While downregulation of H3K9me2 and cytosine methylation of DNA by CM-272 treatments prolonged the survival of mice engrafted with human leukemia and lymphoma cell lines [98], this inhibitor also triggered an immunogenic-based regression of bladder tumors and metastasis [73]. Such an observation is in line with other findings that point toward the suppression of CSC activity to explain the restoration of immune sensitivity in tumors. Furthermore, combinatorial inhibition of G9a activity together with other HMTases such as EZH2 yielded enhanced growth inhibition in vitro and suppression of tumorigenesis in preclinical in vivo models [99, 100]. This represents an important concept to enhance future clinical strategies by encompassing multiple aspects of intratumor heterogeneity, including different subpopulations of resident CSCs.

Recently, a new aminoindole derivative structure (DS79932728) was identified as a potent and orally bioavailable inhibitor of G9a activity (Table 1) [101]. Although no anticancer effects were yet reported for this small molecule, it induced reexpression of γ-globin production in primate in vivo models of β-thalassemia and sickle cell anemia [101]. Thus, DS79932728 currently represents the direct inhibitor of G9a with the highest translational potential for future therapeutic applications.

### Challenges posed by direct targeting of G9a in cancer stem cells

Despite the significant preclinical success demonstrated by targeting G9a activity using direct binding inhibitors, there remain no reports of clinical trials using these or other known G9a inhibitors. One potential explanation to this could reside in substantial toxicity on longer-term functions of G9a necessary for somatic tissue homeostasis. Akin to its role in regulating cell fate during early development, G9a is also essential to maintain stem/progenitor populations and specific normal lineages in healthy tissues (Fig. 2) [48, 102, 103]. Thus, genetic ablation or direct pharmacological suppression of G9a activity have led to different adverse effects. Specifically, liver-specific G9a knockout mice demonstrate significant deleterious effects in tissue maturation, lipid metabolism, and inflammatory responses [104]. Moreover, conditional deletion of G9a in mouse models shows that G9a is required for regulating homeostasis in cardiomyocytes of the adult heart [105]. Alternatively, conditional knockout of G9a in the murine hematopoietic system reveals a failure in T helper cell differentiation, resulting in an impaired immunological response to common gastrointestinal parasite infections [106]. Ugarte et al. also showed that G9a inhibition using UNC0638 delayed normal hematopoietic stem/progenitor cell differentiation in vitro [48]. Taken together, these findings may explain the paucity of in vivo investigations and the lack of clinical trials using these compounds. As a result, it is likely that upstream, context-specific mechanisms regulating G9a may represent an optimal strategy to develop translational tools targeting the epigenetic signature of CSCs in the clinic.

## Harnessing phenotypic screening approaches for advanced G9a-focussed drug discovery

**Fig. 3:**
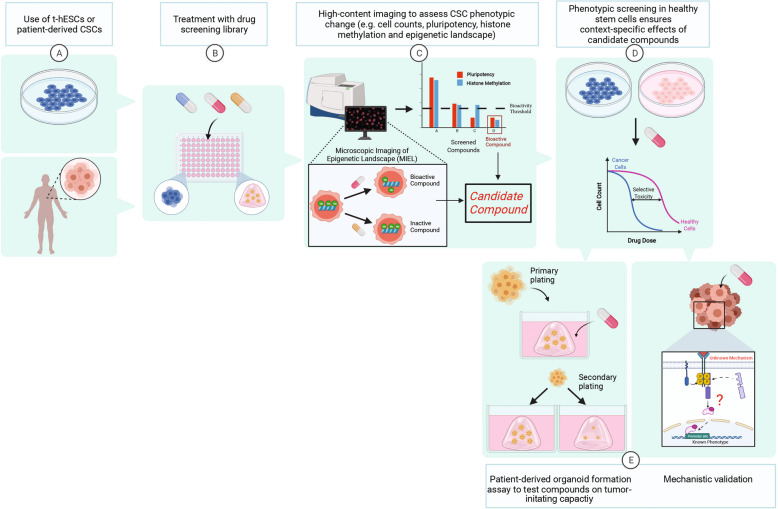
Potential phenotypic screening approach to identify CSC-specific inhibitors of G9a. High-content imaging and Microscopic Imaging of Epigenetic Landscape (MIEL) analysis on t-hESCs or patient-derived CSCs identify compounds that induce key phenotypes (e.g., loss of H3K9me2 deposition and/or OCT4 expression). The application of a cancer-selectivity screening step (or filter) involving CSCs vs. their healthy counterparts enables the exclusion of noncancer-specific compounds. In parallel with mechanistic validation experiments, low-to-medium throughput screening for molecular candidates restricting self-renewal and tumor-initiating activity can be performed using patient-derived serial organoid plating assays.

Most of the reported G9a inhibitors originate from target-centric chemical screening approaches and lead optimization strategies such as structure-activity relationship (SAR) studies [95]. Although high-quality direct inhibitors of G9a are currently available, it appears that global, systemic inhibition approaches targeting H3K9me2 deposition are not suitable for clinical application. Therefore, it is possible that G9a functions in cancer must be targeted via context-specific—or cancer-specific—mechanisms to emerge as a clinically-safe and effective therapeutic strategy. Context-specific EZH2 inhibitors were previously developed to selectively bind mutated forms of the enzyme driving oncogenic deposition of H3K27me3 in lymphoma [107, 108]. One of these inhibitors (tazemetostat) recently received FDA approval to treat refractory follicular lymphoma and epithelioid sarcoma [109]. Considering newly uncovered recurrent G9a mutations in melanoma, it is conceivable that mutant-specific small molecules could be developed to selectively target H3K9me2 deposition in tumor cells while having a limited impact on healthy tissues. Phenotypic drug screening (PDS) represents another strategy to identify compounds that induce specific biological effects (i.e. phenotypes) in whole-cell models (Fig. 3) [110]. PDS was acknowledged to capture drug candidates with pathologically relevant mechanisms of action, conferring higher chances of success for lead compounds at later stages of clinical testing [111, 112]. PDS, however, requires considerable investment in time and resources to identify the target(s) of candidate compounds and to characterize the mechanism of action [112]. To mitigate such an aspect of PDS, it is possible to delineate and multiplex robust readouts in cell-based screening assays, in order to identify compounds affecting recognized pathologically relevant targets and molecular mechanisms. This concept, known as mechanism-informed phenotypic screening, was made easier with the emergence of high-content imaging technologies enabling high-throughput assessment of cell parameters simultaneously in response to treatments (Fig. 3) [110, 113].

A notable example of PDS that led to the identification of a context-specific, CSC-targeting compound was reported by Sachlos et al., using a transformed variant of human ES cells (t-hESCs) as a surrogate model of neoplastic stemness [114]. With respect to shared transcriptional and epigenetic networks between early development and neoplastic stemness, t-hESCs are well-documented as being predictive of drug responses in human CSCs from different origins [6, 21, 115, 116]. By monitoring fluctuations in OCT4 promoter activity in t-hESCs following treatments with clinically approved compounds, the authors identified thioridazine, an antipsychotic drug that primarily antagonizes the dopamine receptor D2 (DRD2), as a novel leukemic stem cell targeting agent in vitro and in vivo [114]. DRD2 was identified as a new context-specific target in self-renewing leukemic progenitors and represents an attractive therapeutic network to eliminate CSCs without impacting normal hematopoietic stem cells [117]. Another instance of context-specific pathway and drug candidate identification in CSCs using PDS on t-hESCs was recently reported using natural product extracts from microorganisms [6]. In this case, a high-throughput screen measuring cell count variations in t-hESCs, and healthy ES cells identified McM025044 as a new SUMOylation inhibitor showing selective toxicity in primary AML CSCs vs. normal hematopoietic progenitors [6]. This reinforces the relevance of pathological networks shared between CSCs from somatic cancers and t-hESCs in drug discovery projects, as the SUMOylation pathway was previously documented to be hyperactivated in both, t-hESCs and CSCs from different origins [115, 118]. Interestingly, the cancer-selective inhibition displayed by McM025044 was not observed for other SUMOylation inhibitors such as ML-792, identified via target-centric drug discovery [6]. It is likely that the PDS pipeline used to screen microorganism extracts, which included a cancer-selective filter where only compounds having a significantly higher impact on the viability of t-hESCs over healthy ES cells, led to the exclusion of non-CSC-selective SUMOylation inhibitor candidates.

### PDS as a method to identify novel CSC-specific inhibitors of upstream G9a regulatory pathways

As summarized in previous sections, G9a is participating in multiple layers of chromatin organization as an HMTase and as a protein-protein binding partner of other epigenetic factors. Considering both modes of regulation exerted by G9a in cancer (HMTase and scaffolding functions), the identification of novel inhibitors blocking the expression of G9a via upstream context-specific pathway(s) could represent an attractive strategy to repress its contribution to CSC biogenesis. Of note, multiple studies describing the oncogenic role of G9a are pointing toward its pathological overexpression in multiple types of tumors [21, 52, 73, 90]. PDS has already been shown to hold value for identifying novel inhibitors regulating the endogenous, context-specific expression of factors contributing to the epigenetic signature of CSCs. For instance, Kreso et al. demonstrated the requirement of the PcG member BMI-1 for neoplastic self-renewal activity in human colorectal cancer [33]. BMI-1 is a core member of the Polycomb repressive complex-1 (PRC1), which mediates E3 ubiquitination of histone H2A lysine residues 118 and 119 (H2AK118ub and H2AK119ub), and is frequently overexpressed in cancer [33]. Such chromatin marks are associated with transcriptional silencing at loci marked by PRC2-catalyzed H3K27me3 [119]. A high-throughput PDS strategy based on a BMI-1 luciferase reporter assay was executed to identify compounds decreasing endogenous transcript levels of BMI-1 in HCT116 cells [33]. PTC-209 was identified as a chemical repressor of BMI-1 expression in human tumor cells, at sub-micromolar concentrations. This compound was also shown to block neoplastic self-renewal and tumor-initiating functions of patient-derived colorectal CSCs in vivo, using serial xenotransplantation assays. The authors of this study suggested that PTC-209 represents a targeted approach to suppress features of neoplastic stemness with limited impact on healthy tissues since no changes in digestive function were observed in treated animals [33]. Recently, BMI-1 repression via PTC-209 treatments showed the elimination of CSCs together with an enhanced antitumor immune response in head and neck squamous cell carcinoma in vivo [120]. Thus, the recent success of PTC-209 as a context-specific repressor of BMI-1 sets the stage for additional research on PDS-based identification of endogenous expression inhibitors for other epigenetic factors, such as G9a.

### MIEL as a screening output for epigenetic bioactivity

Since cell-based PDS assays enable the measurement of variations in relative fluorescent intensity upon compound treatments, a method to rapidly identify and characterize chemically-induced changes in epigenetic marks was recently reported by Farhy et al. [121]. Hence, Microscopic Imaging of Epigenetic Landscape (MIEL) relies on high-content imaging and machine-learning techniques to map the unique nuclear profiles associated with alterations in chromatin organization, via immunofluorescent staining (Fig. 3) [121]. This method demonstrates an enhanced ability to detect epigenetic changes, resulting from treatments with bioactive compounds, compared to typical measurements of epigenetic mark intensity using immunoblotting strategies. This represents a significant improvement beyond conventional screening techniques and promises to enhance the effectiveness of future PDS campaigns hunting for viable epigenetically active therapeutics. In the context of G9a, this technique is especially attractive as it allows the identification of new compounds decreasing either its HMTase activity or endogenous expression while monitoring the global impact of molecular candidates on other biological readouts in CSC and healthy stem cell models.

### Limitations of t-hESC-based phenotypic drug screening

While the use of t-hESC and healthy ES models remains promising for PDS, such an approach is not without limitations. Notably, the lack of tissue-specific oncogenic mutations means that this model can fail to identify compounds demonstrating bioactivity towards unique mutational landscapes, in favor of generalized oncogenic molecular networks. As an alternative, miniaturized screening assays using patient-derived tissues grown as organoids was suggested [122]. The use of patient-derived colorectal tumor organoids showed that such an approach effectively captures the unique genetic and epigenetic heterogeneity present in specific cases, which is a better depiction of clinical presentations [122]. Moreover, organoids grown from induced pluripotent stem cells (iPSCs) were collected from patients displaying key genetic determinants (e.g., familial adenomatosis polyposis (colorectal) and KRAS and P53 mutations (ductal pancreatic)) represent a valuable tool for context-specific inhibitor identification [123, 124]. In the context of identifying new CSC-selective G9a inhibitors, the design of a multi-parametric PDS pipeline should be considered. Thus, the compounds selectively inhibiting the deposition of H3K9me2 in t-hESCs over normal ES cells (MIEL-based screening) could be further tested in a patient or iPSC-derived serial tumor organoid plating strategy, mimicking the gold standard in vivo tumor transplantation model for self-renewal and tumor-initiating functions but in a 3D culture setup (Fig. 3) [21]. Still, such an approach does not allow examinations into the contribution of the TME on the maintenance of the CSC phenotype through the participation of cancer-associated fibroblasts, adipocytes, and endothelial cells. Although recent reports describe new screening systems in which underlying stromal cells are cocultured with CSCs in tumor organoids [125], such a strategy represents considerable investments in resources and might not be viable for larger PDS projects.

## Concluding remarks

G9a plays a critical role in neoplastic stemness by promoting CSC self-renewal and tumorigenicity, as well as in mediating interactions with the TME. However, we have seen that the effects of G9a can be context-specific in regard to cancerous vs. healthy stem cell populations and intratumor cell heterogeneity. Due to the likelihood of long-term toxicity on healthy tissues caused by systemic G9a inhibition, novel approaches to develop next-generation G9a inhibitors must be considered. Specifically, campaigns focussing on selectively targeting H3K9me2 deposition in CSCs using mechanism-informed PDS represent promising strategies to uncover highly translational anticancer molecules. Importantly, there are several emerging ES cell-based and patient-derived models that can be harnessed in future PDS projects which may facilitate the finding of such context-specific inhibitors of G9a functions.
